# A comparison of prognoses between surgical resection and radiofrequency ablation therapy for patients with hepatocellular carcinoma and esophagogastric varices

**DOI:** 10.1038/s41598-020-74424-y

**Published:** 2020-10-14

**Authors:** Cheng-Yi Wei, Gar-Yang Chau, Ping-Hsien Chen, Chien-An Liu, Yi-Hsiang Huang, Teh-Ia Huo, Ming-Chih Hou, Han-Chieh Lin, Yu-Hui Su, Jaw-Ching Wu, Chien-Wei Su

**Affiliations:** 1 Division of Gastroenterology and Hepatology, Department of Medicine, Taipei Veterans General Hospital, No.201, Sec. 2, Shipai Rd., Peitou District, Taipei, 11217 Taiwan; 2 Division of General Surgery, Department of Surgery, Taipei Veterans General Hospital, Taipei, Taiwan; 3 Faculty of Medicine, School of Medicine, National Yang-Ming University, Taipei, Taiwan; 4 Endoscopy Center for Diagnosis and Treatment, Taipei Veterans General Hospital, Taipei, Taiwan; 5 Divsion of Gastroenterology and Hepatology, Department of Medicine, West Garden Hospital, Taipei, Taiwan; 6 Department of Radiology, Taipei Veterans General Hospital, Taipei, Taiwan; 7 Institute of Clinical Medicine, School of Medicine, National Yang-Ming University, Taipei, Taiwan; 8 Department and Institute of Pharmacology, School of Medicine, National Yang-Ming University, Taipei, Taiwan; 9 Department of Medical Research, Taipei Veterans General Hospital, Taipei, Taiwan; 10 Department of Accounting, School of Business, Soochow University, Taipei, Taiwan

**Keywords:** Hepatology, Cancer therapy

## Abstract

There has been insufficient investigation of the differences in long-term outcomes between surgical resection (SR) and radiofrequency ablation (RFA) among patients with hepatocellular carcinoma (HCC) and esophagogastric varices (EGV). We retrospectively enrolled 251 patients with treatment-naïve HCC and EGV who underwent SR or RFA as a first-line treatment. Prognostic factors were analyzed using a Cox proportional hazards model. A total of 68 patients underwent SR, and the remaining 183 patients received RFA. Patients who underwent SR were younger, had better liver functional reserves, and had larger tumors. After a median follow-up duration of 45.1 months, 151 patients died. The cumulative 5-year overall survival (OS) rate was significantly higher among patients who underwent SR than those treated with RFA (66.7% vs. 36.8%, *p* < 0.001). Multivariate analysis showed that age > 65 years, multiple tumors, RFA, albumin bilirubin grade > 1, and the occurrence of major peri-procedural morbidity were the independent risk factors that are predictive of poor OS. In conclusion, SR could be recommended as a first-line treatment modality for HCC patients with EGV if the patients are carefully selected and liver function is well preserved.

## Introduction

Hepatocellular carcinoma (HCC) is the second leading cause of cancer death among males and the sixth among females^1^. Worldwide, it is estimated that around 745,000 patients die of HCC annually^1^. Vaccination programs for hepatitis B virus (HBV) have been successfully implemented for newborns, and antiviral therapy is widely prescribed for chronic HBV or hepatitis C virus (HCV) infection. Thus, the incidence and mortality of HCC have seemed to decline in several traditionally high-risk countries in East Asia^2,3^. Nevertheless, the prevalence rates of HCC are still rising in North America and Europe, especially among the elderly^3,4^. In the United States, the age-adjusted annual incidence rate of HCC increased from 4.4 to 6.7 per 100,000 individuals between 2000 and 2012^4^. Moreover, the prevalence of HCC for patients on a waitlist for liver transplant increased from 6.4% in 2002 to 22.0% in 2017^5^.

With advances in surveillance programs for HCC, more and more patients are diagnosed of HCC at an early stage^6^. For patients with HCC in stage 0 or A in the Barcelona Clinic Liver Cancer (BCLC) system, the recommended curative treatment modalities are liver transplantation, surgical resection (SR), and local ablation therapy according to the current guidelines for HCC management^7^. Among local ablation therapies, percutaneous radiofrequency ablation (RFA) could result in more reliable tumor ablation effects, fewer treatment sessions, lower rates of local recurrence, and higher overall survival (OS) rates than percutaneous ethanol injection therapy^8–10^. Moreover, due to the shortage of organs for liver transplantation, SR and RFA are the most commonly applied therapies for patients with early-stage HCC in daily practice^11^.

Several studies have been conducted to compare the treatment efficacy and prognosis between SR and RFA for patients with early-stage HCC, but the results are inconsistent^12–16^. This might be due to differences in the etiologies, liver functional reserves, and tumor factors among the studies. These factors should be taken into consideration when choosing the optimal treatment modality for patients with early-stage HCC.

Most patients with HCC have an underlying advanced chronic liver disease, such as chronic HBV or HCV infection, alcoholism, or nonalcoholic steatohepatitis^17,18^. With the progression of liver injury and fibrosis, portal hypertension might develop over time. Esophagogastric varices (EGV) start to emerge when the hepatic venous pressure gradient (HVPG) level is greater than 10 mmHg, which is considered to indicate clinically significant portal hypertension (CSPH). This leads to the development of ascites, hepatic encephalopathy, and EGV bleeding with increases in HVPG levels. These conditions are recognized as hepatic decompensation^19,20^. Moreover, CSPH and EGV have been identified as poor prognostic factors for patients with cirrhosis or HCC^18,21,22^. However, the measurement of HVPG levels is costly and not applicable in most hospitals, so EGV has served as a surrogate for CSPH in clinical practice^7,23^.

According to the current international guidelines for the management of HCC, SR is recommended for only patients with a single nodule of any size, good performance status, well-preserved liver function, and normal serum bilirubin levels, as well as a lack of tumor-related symptoms, CSPH, extra-hepatic spread, and major vascular invasion^7^. This is based on several studies that indicate that the presence of CPSH would increase the risk of postoperative liver failure and reduce the OS for HCC patients who undergo SR^22,24,25^.

Instead, local ablation therapy is recommended for HCC patients with CSPH or EGV^7^. Nevertheless, thanks to the recent advances in perioperative management for patients with CSPH, the risk of surgery-associated death has been reduced^26^. Several studies have debated the role the SR in HCC patients with CSPH, and SR could yield acceptable long-term survival in this clinical setting^26–29^. Hence, for patients who have HCC concomitant with EGV, the benefits of SR in comparison to RFA have not been sufficiently investigated^30^. Therefore, we compared the outcomes of SR and RFA in HCC patients with EGV to elucidate this issue.

## Results

### Baseline clinical characteristics

The baseline demographic data of the patients examined are shown in Table 1. Patients who underwent SR were younger than those who received RFA. Both groups were predominantly male, but the ratio of males-to-females was higher in the SR group. Chronic HBV infection was more prevalent in the SR group than the RFA group (58.8% vs. 38.3%, *p* = 0.005). Liver functional reserves were better in the SR group, which had lower scores in the model for end-stage liver disease (MELD), more patients with Child–Pugh Grade A, more patients with albumin bilirubin (ALBI) grade 1, higher serum albumin levels, lower bilirubin levels, lower prothrombin time international normalized ratios (PT INRs), and higher platelet counts. Furthermore, tumor sizes were larger in the SR group than the RFA group (median size: 3.2 cm vs. 2.2 cm, *p* < 0.001).

**Table 1:** Demographics of the study cohort.

Characteristics	All patients	SR	RFA	*p*
	(n = 251)	(n = 68)	(n = 183)	
*Demographic characteristics*				
Age (years)	67 (59–76)	64 (54.5–70)	70 (61–78)	< 0.001
Sex (Male) (%)	167 (66.5%)	57 (83.8%)	110 (60.1%)	0.001
HBsAg (+/−) (%)	110/141 (43.8/56.2%)	40/28 (58.8/41.2%)	70/113 (38.3/61.7%)	0.005
Anti-HCV (+/ −) (%)	111/140 (44.2/55.8%)	26/42 (38.2/61.8%)	85/98 (46.4/53.6%)	0.307
*Liver functional reserve and biochemistry tests*				
MELD Score	9.02 (7.80–11.0)	7.89 (6.98–9.43)	9.5 (8–11.75)	< 0.001
Child–Pugh class (A/B)(%)	206/44 (82.1/17.9%)	67/1 (98.5/1.5%)	139/44 (76/24%)	< 0.001
ALBI (1/2/3) (%)	62/174/15 (24.7/69.3/6%)	30/36/2 (44.1/52.9/2.9%)	32/138/13 (17.5/75.4/7.1%)	< 0.001
Albumin (g/dL)	3.5 (3.2–4.0)	3.8 (3.6–4.2)	3.4 (3.1–3.9)	< 0.001
ALT (U/L)	45 (28–73)	48 (27–87)	43 (28–71)	0.092
AST (U/L)	54 (35–83)	48 (28–84)	55 (37–83)	0.554
ALK-P (U/L)	99 (73–124)	99 (70–121.5)	101 (78.5–126)	0.132
γGT (U/L)	59 (34–102)	60 (33.5–101.5)	57.5 (34–103)	0.499
Total bilirubin (mg/dL)	1 (0.7–1.50)	0.75 (0.61–1.14)	1.06 (0.77–1.65)	0.001
Creatinine (mg/dL)	0.89 (0.73–1.11)	0.87 (0.74–1.02)	0.89 (0.72–1.16)	0.225
PT-INR	1.13 (1.05–1.20)	1.06 (1.03–1.16)	1.14 (1.07–1.22)	< 0.001
Platelets (× 1000/mm^3^)	83.5 (59–113)	101.5 (80–166.5)	76 (56–101)	< 0.001
*Tumor factors*				
Tumor size (cm)	2.3 (1.8–3.2)	3.2 (1.88–5.23)	2.2 (1.8–2.8)	< 0.001
Single Tumor (Yes) (%)	193 (76.9%)	52 (76.5%)	141 (77%)	1
AFP	21.5 (7.3–83.4)	21.3 (5.3–162.9)	21.5 (7.9–70.7)	0.059
*EGV factors*				
EV grade (F1without RCS/F1 with RCS/F2/F3)	86/28/104/31 (34.5/11.1/41.8/12.4%)	30/8/25/5 (44.1/11.8/36.8/7.4%)	56/20/79/26 (30.9/11.0/43.6/14.4%)	0.162
High risk EV (+/−) (%)	163/88 (64.9%/35.1%)	38/30 (55.9%/44.1%)	125/58 (68.3%/31.7%)	0.092
Presence of GV (+/−) (%)	42/209 (16.7%/83.3%)	9/59 (13.2%/86.8%)	33/150 (18.0%/82.0%)	0.475
EV prophylaxis (+/−) (%)*	131/120 (52.2%/47.8%)	25/43 (36.8%/63.2%)	106/77 (57.9%/42.1%)	0.005
*Peri-procedural morbidity*				
All morbidity (+/−) (%)	40/211 (15.9%/84.1%)	24/44 (35.3%/64.7%)	16/167 (8.7%/91.3%)	< 0.001
Major morbidity (+/−) (%)	15/236 (6.0%/94.0%)	6/62 (8.8%/91.2%)	9/174 (4.9%/95.1%)	0.390

Continuous variables are expressed as median with 25th and 75th percentiles.

*SR* surgical resection; *RFA* radiofrequency ablation; *HBsAg* hepatitis B surface antigen; *HCV* hepatitis C virus; *MELD* Model for End-Stage Liver Disease; *ALBI* Albumin-Bilirubin; *ALT* Alanine aminotransferase; *AST* Aspartate aminotransferase; *ALK-P* Alkaline phosphatase; *γGT* γ-Glutamyl transpeptidase; *PT-INR* prothrombin time international normalized ratio; *AFP* alpha-fetoprotein; *EGV* esophagogastric varices; *EV* esophageal varices; *RCS* red color sign; *GV* gastric varices.

*The were 23 (60.5%) and 92 (73.6%) patients with high risk EVs who had EV prophylaxis in SR and RFA groups, respectively (*p* = 0.179).

There were 251 patients who had EGV that was confirmed by an esophagogastroduodenoscopy (EGD). Regarding EGV status, 209 of these patients had esophageal varices (EV) alone at the time of HCC diagnosis, while 2 patients had gastric varices (GV) alone. The remaining 40 patients had both EV and GV. Moreover, 131 (52.2%) patients received prophylaxis therapy for EV bleeding, including 21 patients with non-selective beta-blockers (NSBB), 75 patients with esophageal variceal ligation (EVL) therapy and 35 patients with NSBB and EVL combination therapy.

Compared to the SR group, the RFA group had more cases of high-risk EV (68.3% vs. 55.9%, *p* = 0.092), and more patients received prophylaxis therapy for EV bleeding (57.9% vs. 36.8%, *p* = 0.005). However, among those who had high-risk varices, 23 patients (60.5%) patients in the SR group received prophylaxis therapy, while 92 patients (73.6%) received it in the RFA group (*p* = 0.179).

### The safety of SR and RFA in HCC patients with EGV

No patients in our cohort died during the operations, both the SR group and the RFA group. As shown in Tables 1 and 2, there were 40 patients (15.9%) who developed peri-procedural morbidity and 15 patients (6.0%) who had major morbidity. The SR group had more peri-procedural morbidity than the RFA group (35.3% vs. 8.7%, *p* < 0.001), but the rates of major morbidity were comparable between both groups (8.8% vs. 4.9%, *p* = 0.390). The 90-day mortality rates were 2.9% and 1.1% in the SR group and RFA group, respectively (*p* = 0.371).

**Table 2:** Peri-procedural morbidities of HCC patients who underwent SR or RFA.

	SR, No. (%)	RFA, No. (%)
Overall morbidity	24 (35.3%)	16 (8.7%)
Major morbidity	6 (8.8%)	9 (4.9%)
Coronary artery disease	0 (0%)	0 (0%)
Cerebral vascular accident	0 (0%)	0 (0%)
Postoperative hemorrhage	0 (0%)	1 (0.5%)
Esophageal varices bleeding	1 (1.5%)	3 (1.6%)
Ascites	5 (7.4%)	2 (1.1%)
Hemothorax	3 (4.4%)	2 (1.1%)
Bile leakage	6 (8.8%)	0 (0%)
Infectious complications	6 (8.8%)*	2 (0.5%)**
Deterioration in liver function	11 (16.2%)	4 (2.2%)
Post-operative liver failure	2 (2.9%)	2 (1.1%)
Renal failure	0 (0%)	0 (0%)
Respiratory failure	1 (1.5%)	0 (0%)
Post-operative fever	23 (33.8%)	22 (12.0%)

Major morbidity included post-operative liver failure, postoperative hemorrhage with hematoma formation, esophageal varices bleeding, abscess required drainage, bile leakage required drainage, and respiratory failure.

*HCC* hepatocellular carcinoma; *SR* surgical resection; *RFA* radiofrequency ablation.

*1 intrabdominal abscess, 1 urinary tract infection and 1 surgical site infection in the SR group.

**1 intraabdominal abscess and 1 urinary tract infection in the RFA group.

Among the 68 patients in the SR group, 63 patients underwent conventional open liver resection (OLR), including 15 patients with major resection (defined as resection of three or more segments). Among the 5 patients who underwent laparoscopic liver resection (LLR), 2 patients had two segmentectomies, and the other 3 patients had one segmentectomy. The patients who underwent major resection had more major morbidity than those who received minor hepatectomy (26.7% vs. 3.8%, *p* = 0.025).

### Factors associated with OS

**Figure 1:**
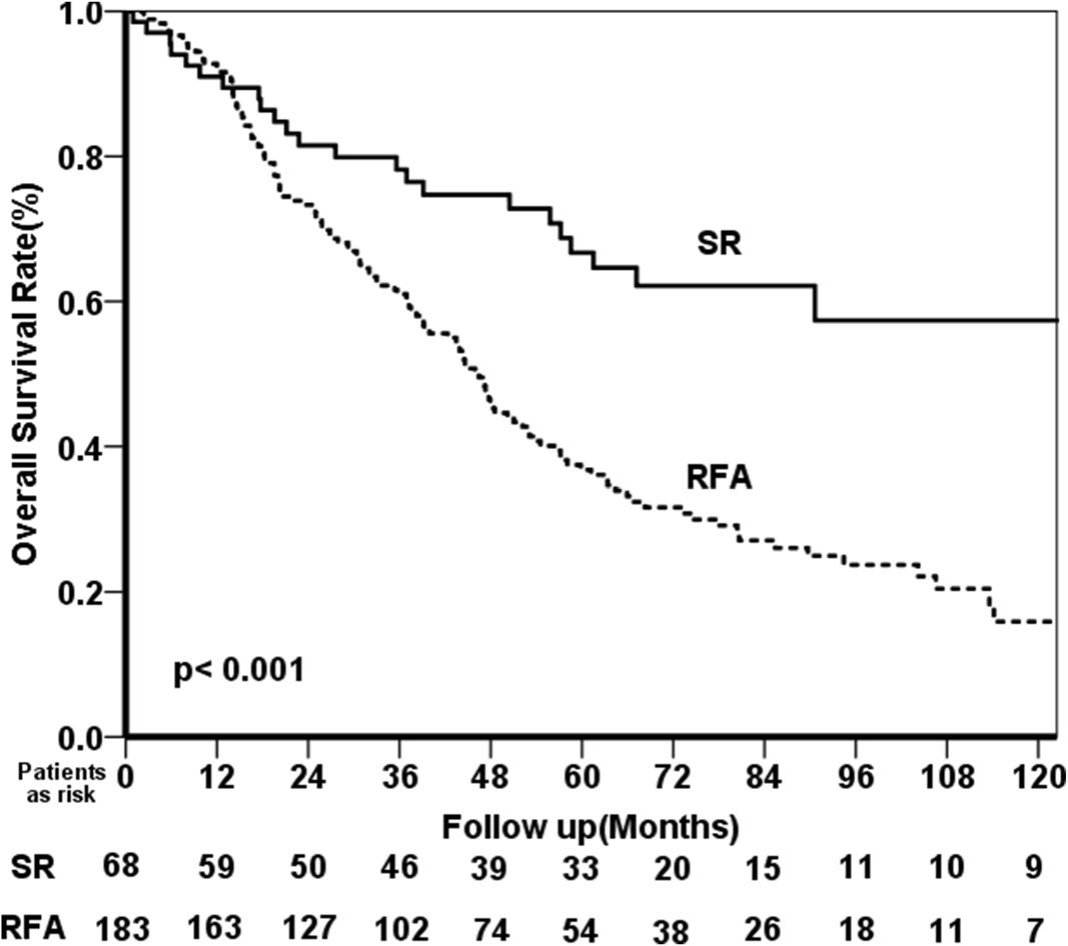
Comparison of the OS rates between HCC patients with EGV who received SR and RFA as a primary treatment modality.

After a median follow-up duration of 45.1 (months interquartile range (IQR): 19.6–69.8 months), 151 patients died. In the SR group vs. the RFA group, the cumulative OS rates at 1, 2, 3, 5, and 10 years were 91.0% vs. 92.2%, 81.5% vs. 73.3%, 78.2% vs. 61.0, 66.7% vs. 36.8%, and 57.4% vs. 15.9%, respectively (Fig. 1; *p* < 0.001). As shown in Table 3, the multivariate analysis revealed that the independent risk factors for poorer OS were age > 65 years (hazard ratio (HR): 1.721; 95% confidence interval (CI): 1.213–2.441; *p* = 0.002), having multiple tumors (HR: 1.630; 95% CI: 1.129–2.354; *p* = 0.009), RFA (HR: 2.271; 95% CI: 1.427–3.616; *p* = 0.001), ALBI grade > 1 (HR: 1.583; 95% CI: 1.032–2.427; *p* = 0.035), and the development of major peri-procedure morbidity (HR: 3.201; 95% CI: 1.774–5.777; *p* < 0.001).

**Table 3:** Analysis of factors associated with poor OS.

Parameters		Univariate analysis			Multivariate analysis		
		HR	95% CI	*p*	HR	95% CI	p
Age (years)	> 65 vs.≦65	2.129	1.515–2.991	** < 0.001**	**1.721**	**1.213–2.441**	**0.002**
Sex	Male vs. Female	0.993	0.708–1.392	0.968			
HBsAg (+)	No vs. Yes	1.808	1.292–2.532	**0.001**			
Anti-HCV (+)	Yes vs. No	1.104	0.801–1.521	0.547			
Albumin (g/dL)	≦3.5 vs. > 3.5	1.972	1.421–2.737	** < 0.001**			
Bilirubin (mg/dL)	> 1.6 vs.≦1.6	1.481	1.034–2.122	**0.032**			
ALT (U/L)	> 40 vs.≦40	0.852	0.616–1.178	0.334			
ALK-P (U/L)	> 100 vs.≦100	0.978	0.678–1.412	0.907			
PT-INR	> 1.1 vs.≦1.1	1.232	0.890–1.705	0.209			
AFP (ng/ml)	> 20 vs.≦20	1.217	0.877–1.690	0.241			
Multiple Tumors	Yes vs. No	1.549	1.076–2.230	**0.019**	**1.630**	**1.129–2.354**	**0.009**
Tumor size (cm)	> 3 vs.≦3	0.784	0.538–1.142	0.205			
Treatment modality	RFA vs. SR	2.658	1.698–4.159	** < 0.001**	**2.271**	**1.427–3.616**	**0.001**
PLT (× 1000/mm^3^)	≦100 vs. > 100	1.389	0.975–1.980	**0.069**			
ALBI grade	2 + 3 vs. 1	1.920	1.270–2.903	**0.002**	**1.583**	**1.032–2.427**	**0.035**
All peri-procedural morbidity	Yes vs. No	1.250	0.802–1.950	0.324			
Major morbidity	Yes vs. No	3.298	1.859–5.851	** < 0.001**	**3.201**	**1.774–5.777**	** < 0.001**
High risk EV	Yes vs. No	1.395	0.984–1.979	**0.062**			
Presence of GV	Yes vs. No	1.449	0.974–2.157	**0.067**			
EV prophylaxis	No vs Yes	1.362	0.988–1.876	**0.059**			

*HR* hazard ratio; *CI* confidence interval; *HBsAg* hepatitis B surface antigen; *HCV* hepatitis C virus; *ALT* alanine aminotransferase; *ALK-P* alkaline phosphate; *PT INR* prothrombin time international normalized ratio; *AFP* alpha-fetoprotein; *RFA* radiofrequency ablation; *SR* surgical resection; *PLT* platelet; *ALBI* Albumin-Bilirubin.

### Risk factors associated with tumor recurrence

**Figure 2:**
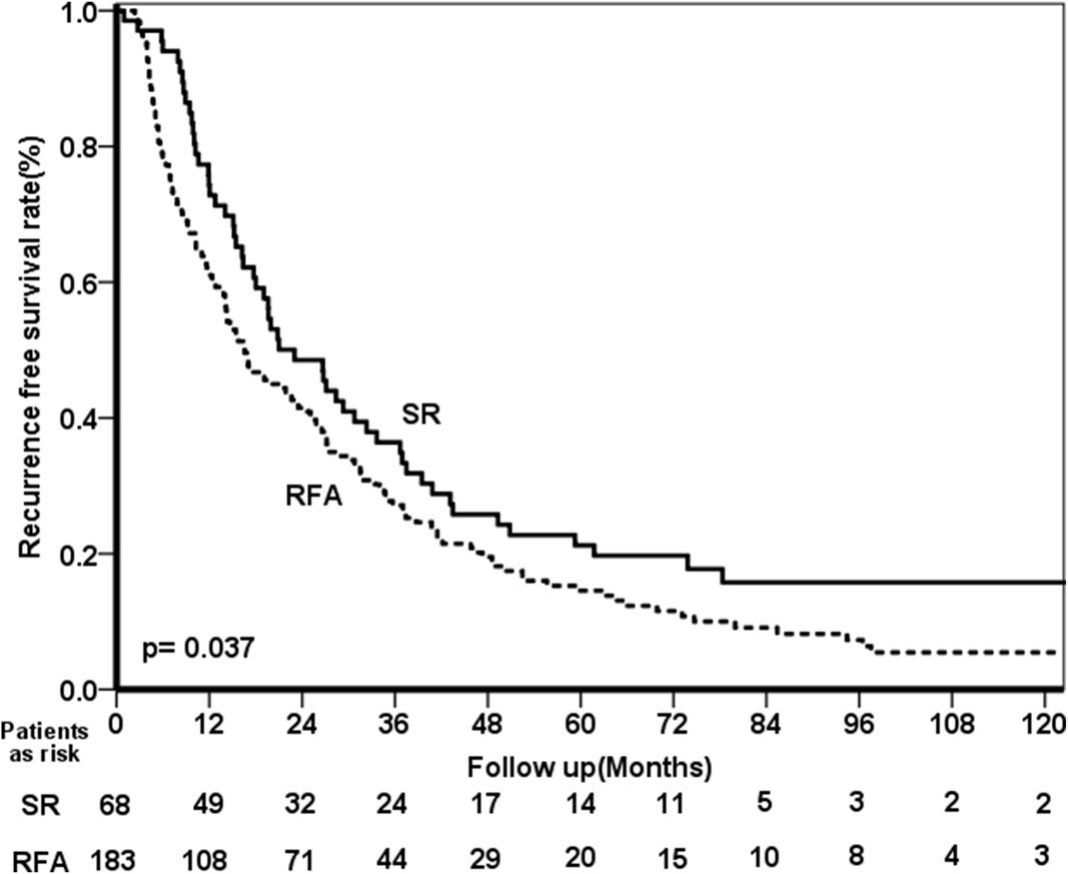
Comparison of the recurrence-free survival rates between HCC patients with EGV who received SR and RFA as a primary treatment modality.

After therapy, 169 patients developed tumor recurrence, and the median recurrence time was 15.2 (IQR 7.3–31.5) months. In the SR group, 49 patients had tumor recurrence with a median development time of 20.8 (IQR 12.4–38.5) months. Among patients who underwent RFA, 120 of them developed tumor recurrence within a median time of 11.9 (IQR 5.4–27.2) months. Patients who underwent SR had a significantly higher rate of recurrence-free survival (RFS) than who received RFA (Fig. 2). As shown in the Table 4, the multivariate analysis showed that multiple tumors (HR 1.421, 95% CI 1.002–2.016, *p* = 0.049) and RFA (HR 1.583, 95% CI 1.128–2.221, *p* = 0.008) were associated with higher recurrence rates after therapy.

**Table 4:** Analysis of factors associated with tumor recurrence rate.

Parameters		Univariate			Multivariate		
		HR	95% CI	*p*	HR	95% CI	*p*
Age (years)	> 65 vs.≦65	1.318	0.970–1.791	**0.077**			
Sex	Male vs. Female	1.101	0.795–1.526	0.563			
HBsAg (+)	No vs. Yes	1.282	0.944–1.739	0.111			
Anti-HCV (+)	Yes vs. No	1.163	0.856–1.580	0.334			
Albumin (g/dL)	≦ 3.5 vs. > 3.5	1.437	1.058–1.951	**0.02**			
Bilirubin (mg/dL)	> 1.6 vs.≦1.6	1.333	0.907–1.958	0.143			
ALT (U/L)	> 40 vs.≦40	0.960	0.705–1.306	0.794			
ALK-P (U/L)	> 100 vs.≦100	0.982	0.697–1.385	0.919			
PT-INR	> 1.1 vs.≦1.1	1.400	1.022–1.918	**0.036**			
AFP (ng/ml)	> 20 vs.≦20	1.089	0.801–1.482	0.585			
Multiple Tumors	Yes vs. No	1.425	1.005–2.019	**0.047**	**1.421**	**1.002–2.016**	**0.049**
Tumor size (cm)	> 3 vs.≦3	0.946	0.678–1.321	0.746			
Treatment modality	RFA vs. SR	1.586	1.130–2.225	**0.008**	**1.583**	**1.128–2.221**	**0.008**
PLT (× 1000/mm^3^)	≦ 100 vs. > 100	0.915	0.658–1.273	0.599			
ALBI grade	2 + 3 vs. 1	1.406	1.001–1.974	0.049			
All peri-procedural morbidity	Yes vs. No	1.229	0.748–2.017	0.416			
Major morbidity	Yes vs. No	1.611	0.659–3.939	0.296			
High risk EV	Yes vs. No	0.930	0.680–1.274	0.653			
Presence of GV	Yes vs. No	1.216	0.823–1.795	0.326			
EV prophylaxis	No vs Yes	0.951	0.701–1.289	0.746			

*HR* hazard ratio; *CI* confidence interval; *HBsAg* hepatitis B surface antigen; *HCV* hepatitis C virus; *ALT* alanine aminotransferase; *ALK-P* alkaline phosphate; *PT INR* prothrombin time international normalized ratio; *AFP* alpha-fetoprotein; *RFA* radiofrequency ablation; *SR* surgical resection; *PLT* platelet; *ALBI* Albumin-Bilirubin; *EV grade*: Esophageal varices grade: F1-RCS: F1 without red color sign; *F1* + *RCS* F1 with red color sign; *GV* gastric varices.

## Discussion

This study shows that SR could provide acceptable long-term outcomes for patients with HCC and EGV. The 10-year cumulative OS rates were 57.4% and 15.9% for patients who underwent SR and RFA, respectively. The survival benefits of SR over RFA were confirmed by the multivariate analysis. Moreover, SR could provide a lower rate of recurrence and a higher RFS rate than RFA. This indicates that SR is not contraindicated for HCC patients with EGV. On the contrary, it could have a survival advantage over RFA if patients are carefully selected.

The presence of EGV, is a surrogate for CSPH and has been validated as an independent factor for poor prognosis among patients with HCC^18,31,32^. Several studies show that the incidence of developing liver decompensation after SR is high among HCC patients with CSPH, which would increase the risk of mortality^24,25,33^ (Table 5). Consequently, it has been suggested that SR be reserved for patients without CSPH in the current guidelines for the management of HCC, whereas RFA is recommended for HCC patients with CSPH^23,34^.

**Table 5:** Summary of the impact of CSPH and EV on the outcomes of patients with HCC after SR.

First author (published year)	Study design	Summary	Reference number
Bruix (1996)	Single-center retrospective cohort study in Spain	1. Among the 29 HCC patients with Child–Pugh class A cirrhosis and underwent SR, 11 patients developed unresolved liver decompensation 3 months after the operation 2. HVPG was associated with the occurrence of unresolved decompensation (OR: 1.90, 95% CI: 1.12–3.22, *p* = 0.0001)	^22^
Llovet (1999)	Single-center retrospective cohort study in Spain	1. This study enrolled 164 cirrhotic patients with HCC, including 77 patients underwent SR and 87 patients underwent liver transplantation 2. CSPH was associated with poor OS for patients who underwent SR by a multivariate analysis (OR: 3.6, 95% CI: 1.4–9.2, *p* = 0.006)	^24^
Ishizawa (2008)	Single-center retrospective cohort study in Japan	1. Among the 386 HCC patients with available records of the status of PHT, 136 patients with PHT and 250 patients without PHT at the time of SR 2. The 5-year OS rates were lower in patients with PHT compared to those without PHT in patients with Child–Pugh class A cirrhosis (56% vs. 71%, *p* = 0.008) 3. However, the status of PHT was not associated with OS and recurrence by multivariate analyses	^26^
Torzilli (2013)	International multicenter retrospective cohort study in 10 hospitals (3 in Asia, 3 in America and 4 in Europe)	1. Among the 2046 consecutive HCC patients who underwent SR, 1883 patients had a record of EV status, including 196 patients with EV and 1687 patients without EV 2. The 5-year OS rates were significantly lower in patients with EV compared to those without EV (44% vs. 59%) 3. A multivariate analysis confirmed that EV was an independent risk associated with poor OS (HR 2.18, 95% CI: 1.48–3.21, *p* < 0.001)	^28^
Berzigotti (2015)	Meta-analysis	1. Eleven studies including a total of 1737 patients who underwent SR for HCC were enrolled for the final meta-analysis 2. The presence of CSPH increased the risk of 5-year mortality (OR: 2.07, 95% CI: 1.51–2.84) and postoperative clinical decompensation (OR: 3.04, 95% CI: 2.02–4.59) versus absence of CSPH	^25^
Vitale (2015)	A nationwide retrospective cohort study in Italy	1. Among the 2090 BCLC stage A-C HCC patients, 550 patients underwent SR, 1046 patients received local regional therapy, and 494 patients received best supportive treatment 2. The advantage of SR in OS was persistent across different tumor stages and CSPH statuses, but not patients with a MELD score > 9, Child–Pugh class B, or performance status > 1	^27^
Qiu (2015)	Single-center retrospective cohort study in China	1. Among 259 patients with HBV-related HCC within the Milan criteria and with portal hypertension, 123 patients underwent SR and 136 underwent ablation 2. Compared to those who received ablation patients who underwent SR had more grade I complications by Clavien-Dindo system, but not for grade II-IV complications 3. The RFS (HR 1.582, 95% CI: 1.222–2.155, *p* = 0.001) and OS (HR 1.739, 95% CI 1.209–2.494, *p* = 0.005) rates were lower in the ablation group than in the SR group confirmed by a multivariate analysis 4. The survival benefit of SR over ablation was still observed after PSM analysis	^30^
Harada (2015)	Retrospective cohort study in two hospitals in Japan	1. Among the 502 HCC patients who underwent SR, 100 with EV and 402 without EV 2. The 5-year RFS rates were comparable (29.6% in EV group vs. 30.3% in non-EV group, *p* = 0.906) between the two groups of patients 3. The 5-year OS rates were higher in patients without EV than those with EV (67.2% in non-EV group vs. 44.9% in EV group, *p* = 0.003). However, EV was not associated with poorer OS after a multivariate analysis 4. The OS rates were similar between patients without EV and those with EV but had an indocyanine green retention test at 15 min > 17%	^36^
Roayaie (2015)	International multicenter retrospective cohort study in 20 hospitals	1. This study included 8656 patients from Asia–Pacific, European, and North American regions 2. For patients who were not ideal candidates for SR (multiple tumors or presence of PHT), SR still provided a better OS than other treatment modalities 3. For patients underwent SR, PH was not an independent risk factor associated with poor OS after resection (HR: 1.170, 95% CI: 0.959–1.427, *p* = 0.123)	^37^
Cucchetti (2016)	Single-center prospective cohort study in Italy	1. This study prospective enrolled 70 consecutive HCC patients undergoing SR in Italy. Among them, 34 (48.6%) patients had an HVPG ≥ 10 mmHg 2. Patients with a higher HVPG level had a higher risk of PHLF compared to their counterparts 3. For patients with HVPG level ≥ 10 mmHg but with MELD score < 10 mmHg, the rate of grade B/C PHLF was only 14.3% if they underwent wedge resections	^33^
Chang (2018)	Single-center retrospective cohort study in Taiwan	1. Among 446 HCC patients who underwent SR, 89 (20%) had EV 2. The cumulative 5-year OS rates were 62.3 and 70.6% in patients with and without EV, respectively (P = 0.102) 3. EV was not associated with poor prognosis for HCC patients after SR both in terms of OS and recurrence, and it was confirmed by multivariate analyses and PSM	^29^

*CSPH* clinically significant portal hypertension; *EV* esophageal varices; *HCC* hepatocellular carcinoma; *SR* surgical resection; *HVPG* hepatic venous pressure gradient; *OR* odds ratio; *CI* confidence interval; *OS* overall survival; *PHT* portal hypertension; *HR* hazard ratio; *MELD* model for end-stage liver disease; *HBV* hepatitis B virus; *RFS* recurrence-free survival; *PSM* propensity score matching; *PHLF* post-hepatectomy liver failure.

Nevertheless, recent technical innovations in surgical techniques, anesthesia, critical care, and spatial understanding of the intra-hepatic anatomy of the liver have led to an increasing number of liver resections, fewer post-operative hepatic failures, and lower treatment-related mortality^26,35^. As shown in Table 5, several studies from Eastern and Western countries have validated that CSPH alone is not a contraindication for SR^33,36,37^ These findings suggest that the indications for SR could be extended to HCC patients with CSPH or EGV if they have well-preserved liver function.

For patients with early-stage HCC, RFA is relatively safe and has lower costs, less serious adverse effects, and less destruction of non-neoplastic tissue than SR^7^. Moreover, it could provide an acceptable long-term OS for certain patients^38–40^. It has been reported that 5-year accumulative OS rates over 60% could be achieved with RFA among HCC patients with early-stage HCC^12,38–40^. In our previous study, the 5-year and 10-year OS rates after RFA were 63.1% and 48.7%, respectively^40^. Consequently, RFA is regarded as a curative treatment modality for HCC patients^7^.

However, the recurrence rate after RFA is still high. For example, the 10-year RFS rate after RFA was only 12.4% in our previous report^40^. This might be caused by the incomplete ablation of liver tumors due to insufficient ablation-needle technology, tissue cooling by the neighboring blood vessels (through a heat sink effect), large tumor masses, and the ablation of tumors in close proximity to heat-sensitive organs^41^.

In contrast, SR could have a higher chance of complete excision of not only tumor tissue but also the hepatic parenchyma around the tumor, which might have microvascular invasion and micro-metastases^13,42^. Therefore, it could result in better local tumor control than RFA. However, the risk of liver decompensation and mortality after the operation are concerns when performing SR for HCC patients with a poorer liver functional reserve. Several studies compared the outcomes between SR and RFA for patients with early-stage HCC^12–16,43^. Most of these studies demonstrated that SR could reduce the risk of recurrence and might provide superior OS to RFA, although some studies observed that the OS rates were comparable between SR and RFA^12^.

Nevertheless, the differences in prognosis between SR and RFA for HCC patients with CSPH have not yet been investigated sufficiently. Qiu et al. demonstrated that SR is safe and could provide a better OS and RFS than ablation therapy for patients with HBV-related HCC and CSPH^30^. However, the patients enrolled in that study were limited to those with HBV-related HCC, and the ablation therapies included both RFA (79 patients) and microwave ablation (57 patients).

In the current study, we enrolled HCC patients from all etiologies and compared the prognoses between patients who underwent SR and RFA. In the SR group, no patient expired during the surgeries, and only one patient died within one month due to post-operative liver failure. The patients who underwent SR had a higher rate of post-procedure morbidity than those in the RFA group, but the rates of major morbidity and 90-day mortality were not statistically different between the two groups. Notably, our cohort revealed that SR could offer better long-term prognoses than RFA among HCC patients with EGV in terms of OS and recurrence. This was further validated by the multivariate analysis. The results could provide robust evidence for performing SR as a front-line treatment modality for patients with HCC and EGV if they have well-preserved liver function.

The patients in our cohort who underwent RFA as the primary treatment modality were older and had a poorer liver functional reserve than those who underwent SR. Older patients might choose RFA because of the greater chance of comorbidities than younger patients. RFA features less invasiveness, a lower complication rate, and lower costs, as well as higher repeatability in the event of recurrence^44^. This finding is similar to those of a nationwide cohort from Japan^45^. However, the survival benefit of SR over RFA was persistent after adjusting for the confounding factors for prognosis in the multivariate analysis.

**Figure 3:**
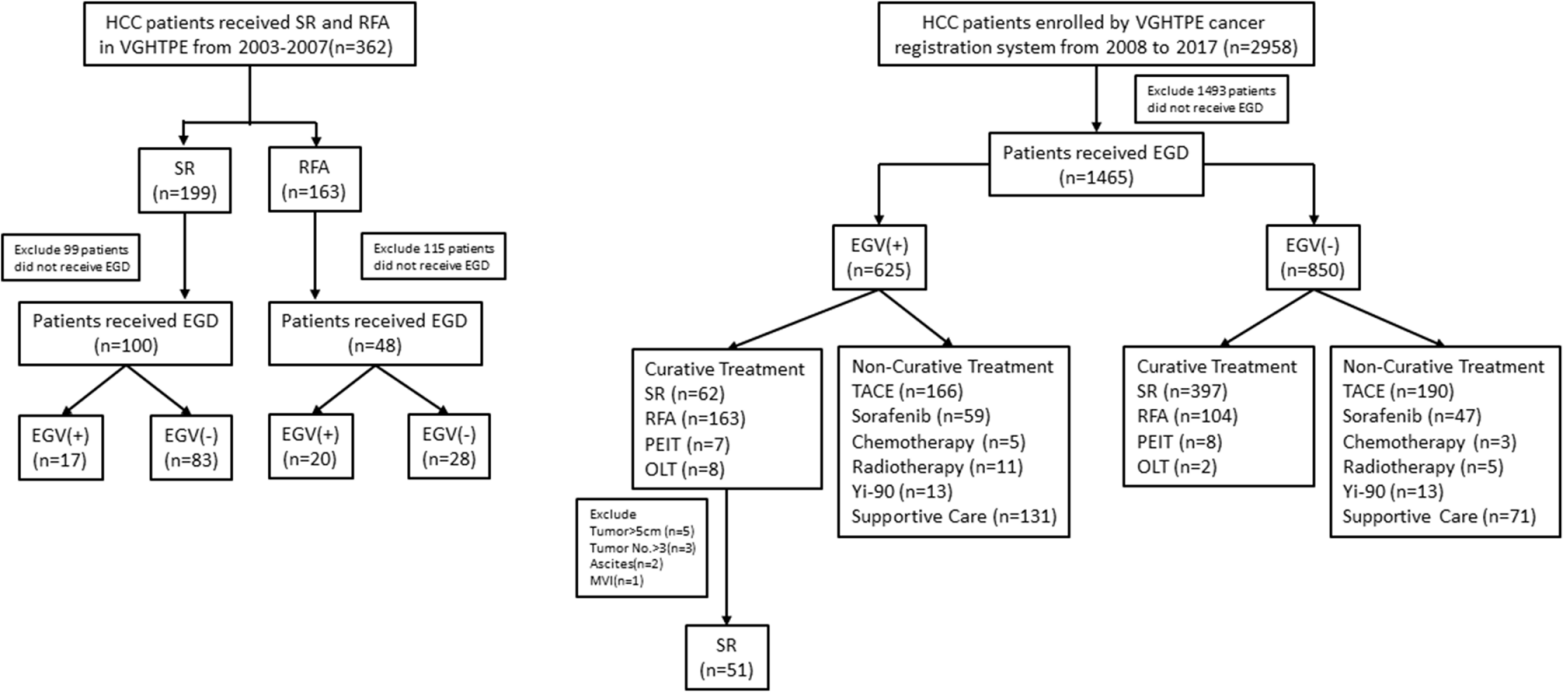
Study flow chart.

There were some limitations to our study. First, although EGD was recommended to screen for EGV among patients with a new diagnosis of HCC, the completion rate was only 48.6% in this cohort (Fig. 3). Selection bias might be present because of the retrospective nature of the study. Second, measuring HVPG levels is the gold standard for assessing the degree of portal hypertension. However, it is invasive, expensive and not feasible in most medical centers. We did not perform HVPG measurement in out cohort. Furthermore, the spleen diameter was not recorded uniformly due to the retrospective study design. Therefore, we used the presence of EGV diagnosed by EGD as a surrogate for CSPH, which is more practical in the daily practice. Third, decisions made for treatment were shared between the physicians and patients. This patient-tailored approach is based on the multidisciplinary evaluation of each case and includes any alternative treatment options. This might have caused the significant demographic difference between the two groups of patients. Fourth, LLR is a recent technical innovation in SR for the treatment of HCC. It could provide shorter hospitalization, less blood loss, less wound pain, and a lower rate of postoperative liver failure and ascites formation than conventional OLR for cirrhotic patients with HCC^46–48^. Regarding the long-term outcomes, HCC patients who underwent LLR had similar OS and RFS rates to those who received OLR^46–49^. However, we could not compare the treatment efficacy and outcomes between LLR and OLR because the majority of patients in our cohort underwent OLR and only 5 patients received LLR. Further prospective studies are warranted to elucidate this issue. Lastly, this study enrolled HCC patients over a relatively long span of time, so the diagnoses, assessment of HCC patients, and SR and RFA techniques might not have been the same between different time periods.

Despite these limitations, this study provides robust evidence to reassure physicians that SR could serve as a first-line treatment option for HCC, in spite of evidence of CSPH and EGV. However, the patients should be selected carefully. Consequently, aggressive treatments beyond the current guidelines could be considered when clinically applicable to achieve the maximum survival benefit.

## Conclusion

SR could be recommended as the first-line treatment modality for HCC patients with EGV if the patients are carefully selected and liver function is well preserved.

## Methods

### Patients

This study retrospectively enrolled 251 treatment-naïve HCC patients who underwent SR or RFA as the first treatment modality for HCC. All of the patients had EGV diagnosed by EGD at the time of HCC diagnosis at Taipei Veterans General Hospital (Fig. 3). EV was classified as follows: F1: small and straight varices; F2: moderately sized, tortuous varices; and F3: large, tumorous varices^50^. High-risk EV was defined by the F2 and F3 classifications or by F1 accompanied by red coloring^51^.

The diagnosis of HCC was established based on the criteria from the American Association for the Study of Liver Disease consensus^52^. Multidisciplinary expert meetings and an HCC registration system in Taipei Veterans General Hospital have been conducted since October 2007. The diagnosis and treatment strategy are discussed at weekly multidisciplinary meetings for all of the HCC patients at this hsopital^53^. The decision about the treatment modality is shared between the patient and the physician after discussing the risks, benefits, complications, and efficacies of the available treatments, as well as the recommendations from the multidisciplinary expert meetings.

In our center, the indications for SR are as follows: (1) Child–Pugh grade of liver function of A or B, with an indocyanine-green 15-min retention rate (ICG-15R) of ≤ 30%; (2) tumor involving no more than two Healey’s segments without involvement of the main portal vein trunk; and (3) the absence of extra-hepatic tumor dissemination^17^. RFA was performed in cases of (1) a single tumor with size < 5 cm or 2‒3 tumors < 3 cm; (2) no extra-hepatic metastasis or major vascular invasion; (3) Child–Pugh grade A or B; (4) platelet count > 50,000 /mm^3^; (5) the absence of ascites; and (6) no other comorbid diseases that might complicate RFA^40^. Liver transplantation was indicated for patients with end-stage liver disease, HCC, or acute liver failure according to the criteria of United Network for Organ Sharing (UNOS)^54^. For patients with HCC, the tumor size criterion for liver transplantation was based on the criteria of the University of California San Francisco (UCSF)^55^.

The indications for SR and RFA were not the same, so we selected HCC patients using the Milan criteria for this study. Consequently, the inclusion criteria were as follows: (1) solitary tumor with size < 5 cm, in which anatomic resection could be achieved after through evaluation or 2‒3 tumors < 3 cm; (2) no extra-hepatic metastasis or major vascular invasion; (3) grade A or B Child–Pugh classification of liver function; (4) platelet count > 50,000 /mm^3^; (5) absence of ascites; and (6) no other comorbid diseases that might complicate SR or RFA (e.g., infection, arrhythmia, acute myocardial infarction, uncontrolled congestive heart failure, chronic obstructive pulmonary disease with acute exacerbation, recent stroke, etc.).

There were 2958 consecutive patients who were enrolled in the HCC registration system database from 2008 to 2017. As shown in Fig. 3, 1465 of these patients received an EGD, and 625 patients had EGV at the time of HCC diagnosis. Patients were excluded for having tumor size > 5 cm, tumor number > 3, ascites or major vascular invasion. After the exclusion, 51 patients were enrolled in the SR group, and 163 patients were enrolled in the RFA group. Moreover, we also retrospectively recruited 17 patients in the SR group and 20 patients in the RFA group who fulfilled the inclusion criteria between 2003 and 2017 before the establishment of the HCC registration system. Consequently, a total of 68 patients who underwent SR and 183 patients who received RFA were enrolled in the final analysis.

After SR or RFA, the peri-procedural morbidities were recorded and graded by the Clavien-Dindo classification^56^. Grade III-V complications were defined as major morbidities. Postoperative liver failure was defined according to the International Study Group of Liver Surgery (ISGLS)^57^. All of the patients were followed up regularly every 3 months after SR or RFA until their last visit to our hospital or death.

This study was conducted in accordance with the Declaration of Helsinki and current ethical guidelines. Approval was obtained from the Institutional Review Board (IRB) of Taipei Veterans General Hospital (VGHIRB No. 2018-07-029BC). Informed consent was obtained before the patients underwent SR or RFA.

### Biochemical and serological markers

Serum biochemistry was measured using a Roche/Hitachi Modular Analytics System (Roche Diagnostics GmbH, Mannheim, Germany). Serum alpha-fetoprotein (AFP) levels were tested using a radioimmunoassay kit (Serono Diagnostic SA, Coinsins, Switzerland). The ALBI score was calculated using the following equation: (– 0.085 × albumin in g/L) + (0.66 × log10 bilirubin in μmol/L)^58^. The ALBI grades were defined as grade 1 (score ≤ – 2.60), grade 2 (score >  − 2.60 and ≤ – 1.39), or grade 3 (score > – 1.39).

### Statistical analyses

The primary endpoint of the study was OS, which was calculated from the date of HCC diagnosis until death, the last visit, or loss to follow-up. Pearson’s chi-squared analysis or Fisher’s exact test was used to compare categorical variables. The medians with IQRs were used to express continuous variables and compared using the Mann–Whitney U test.

Cumulative rates of OS and RFS were estimated by the Kaplan–Meier method, and the results were compared using a Cox proportional hazards model. Variables that had statistical significance (*p* < 0.05) or were proximate to it (*p* < 0.1) in the univariate analysis were included in a multivariate analysis, which was conducted using a forward stepwise Cox regression model. The ALBI scores were derived from serum albumin and bilirubin levels, so we used the ALBI grade but not the serum albumin and bilirubin levels in the multivariate analysis.

A two-tailed *p* < 0.05 was considered statistically significant. All statistical analyses were performed using IBM SPSS Statistics for Windows, version 21.0 (IBM Corp., Armonk, NY, USA).

## Data Availability

The datasets generated during and analyzed during the current study are available from the corresponding author on reasonable request.
